# Sex Differences in Factors Associated with General Life Satisfaction among Occupationally Injured Workers in Korea: A Panel Analysis of the 2013–2017 Panel Study of Worker’s Compensation Insurance

**DOI:** 10.3390/ijerph16224397

**Published:** 2019-11-11

**Authors:** Jin-Won Noh, Kyoung-Beom Kim, Jooyoung Cheon, Yejin Lee, Young Dae Kwon

**Affiliations:** 1 Department of Health Administration, College of Health Science, Dankook University, Cheonan 31116, Korea; health@dankook.ac.kr; 2 Global Health Unit, Department of Health Sciences, University Medical Centre Groningen, University of Groningen, Groningen 9713 GZ, The Netherlands; 3 Graduate School of Public Health, Korea University, Seoul 02841, Korea; aefile01287@korea.ac.kr; 4 Department of Nursing Science, Sungshin University, Seoul 01133, Korea; jcheon@sungshin.ac.kr; 5 Department of Healthcare Management, Eulji University, Seongnam 13135, Korea; yiye1110@gmail.com; 6 Department of Humanities and Social Medicine, College of Medicine and Catholic Institute for Healthcare Management, The Catholic University of Korea, Seoul 06591, Korea

**Keywords:** Life satisfaction, sex differences, injured workers, worker’s compensation insurance, return to work

## Abstract

The majority of previous occupational studies focused on factors affecting life satisfaction among occupationally injured workers have been based on a cross-sectional design, not a sex-aggregated model. This study aimed to identify sex differences in factors related to life satisfaction among workers who experienced work-related injuries using nationally representative panel data from South Korea. Data from the first to fifth (2013–2017) waves of the Panel Study of Worker’s Compensation Insurance were analyzed. Of 1514 respondents, those who participated in all five survey waves were included in the final study population. To assess the factors associated with general life satisfaction of the occupationally injured workers, a panel data analysis was conducted using generalized estimating equations. The impacts of education level, return to work, self-rated health, task performance, self-esteem, and self-efficacy were significant in both sexes. On the other hand, the influence of age, marital status, personal labor income, and National Basic Livelihood Act recipient status significantly varied by sex. There were sex differences in factors related to general life satisfaction among occupationally injured workers, highlighting the need for sex-specific intervention programs. Employers, healthcare providers, and other stakeholders need to pay attention to vulnerable groups and investigate the most appropriate financial support.

## 1. Introduction

The International Labor Organization (ILO) and World Health Organization (WHO) estimate occupational injuries and illnesses or burden of disease. The ILO has made global estimates from the point of view of occupational burden and the WHO from an overall health point of view. Both have concluded 5–7% of all fatalities in industrial countries are attributed to work-related illness or occupational injury [1,2]. The WHO recently estimated that 20–50% of people are exposed to various hazards at work worldwide, and this proportion is likely to be higher in developing and newly industrialized countries [3]. The working population is aging and the economic burden of workplace injuries is increasing in Korea. The economic burden, including indirect loss due to occupational injuries and diseases, was estimated to be more than US $20 billion in 2017, equivalent to 1.31% of the country’s gross domestic product [4].

Life after occupational injuries often differs greatly from life before injury due to residual problems of pain, loss of function, and injury-related concerns about future employability and recurrence [5,6,7]. Previous studies have confirmed the importance of psychosocial factors in explaining levels of life satisfaction after injuries [8,9,10,11,12]. Emotional and psychological aspects have been found to be the best predictors of general quality of life among patients, even considering that their physical factors most affected their chronic conditions [13].

After a life-course disruption such as an occupational injury, it is assumed that people’s perceived life satisfaction will be influenced by ability to function in their desired occupations [14,15]. Everyday occupations become more challenging and more difficult for occupationally injured workers [16]. Reported problems include lack of workplace accommodation, hostility or indifference from supervisors and coworkers, career difficulties, and lack of support for returning to work [17]. Some studies have focused on psychosocial factors influencing duration of disability and return to work after injury [18,19]. Individuals have many negative experiences after a job injury, and those experiences might lead to depression, perceived disabilities, or fear of re-injury [20]. These experiences could affect the length of stay in the hospital or delay return to work.

Female participation in the work force has increased dramatically over the last 30 years. An accompanying trend is that increasing numbers of females are employed in occupations with historically higher injury/illness rates [21]. Sex differences in lives after injury could be occurring not only because of physical differences, but also due to socioeconomic or psychological differences. Among occupationally injured workers, differences in factors related to life satisfaction between men and women have previously been studied [22,23,24,25]. Women with chronic pain showed higher activity level and pain acceptance compared to men [26], and men showed considerably higher incidence of serious traumatic injury outcomes compared to women due to several sex differences [27]. However, only a few studies have presented sex-specific analyses [28,29,30]. Thus, this study aimed to identify sex differences in factors associated with general life satisfaction among workers who experienced work-related injuries using nationally representative panel data from the Republic of Korea.

## 2. Methods

### 2.1. Data and Subjects

This study analyzed data from the first to fifth (2013–2017) waves of the Panel Study of Worker’s Compensation Insurance (PSWCI). The PSWCI is a nationwide interview survey that has been conducted by Korea Workers’ Compensation and Welfare Service since 2013 to investigate the outcomes of care and rehabilitation activities, return to work after occupational accidents, and labor market transitions. The PSWCI’s target population for the years in this study was 89,921 occupationally injured workers who had completed receiving workers’ compensation care in 2012. The target population was restricted to 82,498 after excluding 73 participants who had unknown addressees and 7350 foreigners or residents of Jeju Island. Finally, 2000 workers were extracted using the stratified systematic sampling method according to sex, age, residential region, disability grade, and utilization of a rehabilitation service. This study comprised 1514 respondents who participated in every survey wave, after excluding 486 respondents who did not participate in at least one wave.

This study was approved by the Institutional Review Board of the Catholic University of Korea with a waiver for informed consent (MC18ZESI0095). The PSWCI is an anonymized national public database that is openly accessible at http://www.kcomwel.or.kr/Researchinstitute. There was no identified privacy risk to the study subjects because the data were analyzed with no personally identifiable information.

### 2.2. Variables and Measures

The dependent variable, general life satisfaction, was assessed using six questions on life satisfaction, measured on a five-point Likert scale: household income, leisure life, residential environment, family relationships, relative relationships, and social relationships.

Sociodemographic (age, sex, marital status, and education level), economic (return to work, personal labor income, and National Basic Livelihood Act (NBLA) recipient status), health-related and psychological (self-rated health (SRH), task performance, disability grade, length of hospital stay, self-esteem, and self-efficacy), and time after treatment variables were included in the study model.

Age was classified as ‘29 or younger’, ‘30–39′, ‘40–49′, ‘50–59′, and ‘60 or older’. Marital status was categorized as ‘never married’, ‘divorced, separated, or widowed’, and ‘married’. Education level was classified as ‘middle school graduate or lower’, ‘high school graduate’, and ‘college graduate or higher’. Return to work was categorized into three groups: ‘unemployed’, ‘different workplace’, and ‘pre-injury workplace’. Personal labor income was divided into quartiles for each survey wave and referred only to a respondent’s wage or salary income, excluding financial, insurance, or real estate benefits. A representative basic social assistance law named the National Basic Livelihood Act (NBLA) was enacted in 2000 in South Korea. It aims to protect fundamental human rights for impoverished households that earn less than a designated minimum cost of living per month. Benefiting from the NBLA is commonly considered a proxy measurement of poverty in South Korea. Respondents were categorized as ‘recipients’ or ‘non-recipients’ of benefits from the NBLA.

SRH was measured on a four-point Likert scale of ‘very bad’, ‘bad’, ‘good’, and ‘very good’. Task performance was measured with the following question: “Assume that your task performance is ‘10′ before your occupational injury, how would you rate yourself now?” Responses ranged from ‘0′, representing a complete loss of task performance, to ‘10′. The Industrial Accident Compensation Insurance Act rates industrial disabilities into 1 to 14 grades. Each grade represents profound (grades 1–3), severe (grades 4–7), moderate (grades 8–10), and mild (grades 11–14) disability. In this study, for ease of interpretation, we added a 15th grade that represented no disability. Length of hospital stay was divided into three categories: ‘1 to 364 days’, ‘365 to 729 days’, and ‘730 days or longer’.

Psychological variables included self-esteem and self-efficacy scores. In the PSWCI, the Rosenberg self-esteem scale (RSES) and self-efficacy scale (SES) developed by Sherer et al. were used [31]. The RSES consisted of 10 items, and respondents answered on a four-point Likert scale. The score ranged from 0 to 30, with a score lower than 15 suggesting low self-esteem. Respondents were classified by whether or not they had low self-esteem. The SES was composed of 23 items measured on a five-point Likert scale. Of these items, 13 asked in a negative fashion were reverse scored. The SES measures the general level of confidence in one’s ability. The higher is the score, the greater is the confidence in one’s ability or achievement. To capture the longitudinal effect of elapsed time after completion of workers’ compensation care, time after treatment was employed in the study model (one to five years).

### 2.3. Analytical Approach and Statistics

To summarize the sociodemographic, economic, health-related, and psychological characteristics of occupationally injured workers, a descriptive analysis was performed. The frequency and percentage of qualitative data and the mean and standard deviation (SD) of the quantitative data were reported. To analyze the sex differences in participant characteristics, the two-sample t-test or chi-squared test was conducted by survey wave. A panel data analysis was performed using generalized estimating equations (GEEs) with a log link and exchangeable correlation structure for repeated measurements to assess the factors associated with general life satisfaction of the occupationally injured workers. A two-step approach was employed. The analysis was performed for all participants and then stratified by sex to identify the sex-specific effects. The coefficient and 95% confidence interval (CI) were estimated. The Huber–White sandwich estimator of variance was applied in the GEE model to account for heteroscedasticity and produce robust standard errors. Data were analyzed using the software package Stata/MP 14.2 (StataCorp, College Station, TX, USA). A threshold for statistical significance was set at 5% (two-tailed).

## 3. Results

Table 1 summarizes the general characteristics of occupationally injured workers by survey wave. A total of 7570 observations from 1514 participants in five survey waves were analyzed. Most of the participants were male (82.8%), aged between 50 to 59 years (36.6%), and married (72.6%). About four-fifths of the subjects were high school graduates or lower. About one-third of subjects did not return to work and remained unemployed. Of the entire study population, 1.8% was NBLA recipients. About half of the participants reported a ‘good’ SRH. Most participants were hospitalized for a period between 1 and 364 days (89.6%) and did not show problems of low self-esteem (89.6%). The average level of task performance, self-efficacy, self-esteem, and life satisfaction were lower for females than for males. However, the disability grade was more severe for males than for females (Table 1).

Table 2 presents the results from the GEE model, which investigated the factors associated with general life satisfaction among occupationally injured workers. The influence of age, marital status, personal labor income, and NBLA recipient status varied significantly by sex. Compared to those 29 years old or younger, the older females (aged 50 to 59 years; aged 60 years or older) had higher general life satisfaction, but the older males (aged 40 to 49 years) had lower general life satisfaction. Divorced men had lower general life satisfaction than never married men, and married men had higher general life satisfaction than the never married. Regarding the personal labor income of males, those above the first quartile (second quartile; third quartile; fourth quartile) showed higher general life satisfaction than those in the first quartile. Male NBLA recipients had lower life satisfaction than male non-recipients. In contrast, females did not show differences in general life satisfaction by marital status, personal labor income, or NBLA recipient status. 

The impacts of education level, return to work, SRH, task performance, self-esteem, and self-efficacy were significant in both sexes. Those who had a higher level of education compared to middle school graduates or lower (college or higher among females; high school graduate among males; college or higher among males), those who returned to the workplace (pre-injury workplace among females; different workplace among males; pre-injury workplace among males), those with better SRH compared to very bad (very good among females; bad among males; good among males; very good among males), those who reported a high level of task performance (female; male), and those who showed a higher self-efficacy score (male; female) had higher general life satisfaction. Conversely, those who had a low self-esteem (female; male) had lower general life satisfaction (Table 2).

## 4. Discussion

This study examined multiple factors associated with general life satisfaction among workers who experienced work-related injuries using nationally representative data from the Republic of Korea. In both sexes, age, educational level, return to work, SRH, task performance, self-esteem, and self-efficacy were significant factors associated with general life satisfaction, while marital status, personal labor income, and NBLA beneficiary status were significant factors only for men. Level of disability and length of hospital stay were not significant factors in either sex.

The study findings revealed that occupationally injured Korean men have lower levels of general life satisfaction than occupationally injured Korean women, even though work-related injuries were a critical issue in both sexes. This finding was consistent with a previous study finding that women generally showed higher levels of life satisfaction than men across all socioeconomic status groups after analyzing data from 166 countries [26]. One possible explanation regarding the sex difference is physical and psychological responses to the injury by sex. Women with chronic pain showed a higher activity level and pain acceptance, being less afraid of pain and perceiving higher social support, while men suffered mood disturbances and lower activity levels [32]. A review described that men had considerably bad traumatic injury outcomes compared to women due to anatomical, physiological, immunological, and hormonal differences [27]. Another explanation is that Korean men suffered a greater decrease in income from work-related injuries compared with women and might have feared an unstable social status compared to pre-injury [23,24,33].

Understanding the mechanisms that lie beneath the components of subjective well-being and life satisfaction in middle-aged men is a significant topic [34]. Men in midlife often have more financial responsibilities than other age groups of people and have few financial resources other than employment, whereas younger adults may receive financial support from their parents and older adults may benefit from national and retirement pensions [23]. The finding that recipients of NBLA, a proxy measurement of poverty, showed significantly lower life satisfaction among men but not among women, can be understood in the same context.

Interestingly, the proportion of participants who returned to a different workplace increased five years after injury in both men and women, and the proportions of women who were unemployed and returned to their pre-injury workplace were low compared to those of their male counterparts five years after work-related injuries. The present findings were consistent with previous findings that injured workers were likely to find jobs with a lower income or to become unemployed over time compared to non-injured workers [33,35,36,37], and women were more vulnerable than men in regard to returning to work [25,36].

A novel finding from the current study was the sex differences in general life satisfaction by type of work. Return to pre-injury workplace was associated with better general life satisfaction in both men and women, whereas return to a different workplace was a significant factor in general life satisfaction for men but not for women [38]. For men, return to work itself may indicate proper function as members of society or ability to support their family as a major financial resource, whereas the quality of employment may be more important to women workers’ life satisfaction [22,23,24,25]. Future research should continue to investigate the different impacts of employment status after return to work on subjective well-being among injured men and women.

Marital status was a significant factor in general life satisfaction among injured male workers in this study. Married men showed higher general life satisfaction and men who were divorced, separated, or widowed showed lower general life satisfaction compared to never married men. Married men have more opportunity to receive family support related to their disabilities and psychosocial stress, which may help male workers improve their health and employment outcomes compared to never married men and men who are divorced, separated, or widowed [39,40,41,42]. Also, married men may be more financially stable than men who are divorced, separated, or widowed because 49.3% of all Korean households were dual-earner households in 2017 [43], and this may lead to better general life satisfaction among married men. However, marital status was not a significant factor of general life satisfaction among women. A study reported that marital quality was more important than marital status in life satisfaction among women [41]. Another study stressed the mediating effect of age in quality of life for women because single women in their 30s had the highest quality of life, but married women between 40 and 69 years old showed higher quality of life than single women [42].

Level of disability and length of hospital stay were not significant factors in both sexes. Previous studies have shown that disabled workers after occupational injury were less likely to return to work than the workers without disabilities [44], and economic status of injured workers declined compared to that of pre-injury, regardless of degree of consequences [22,32]. In this study, workers who were disabled may be threatened by income changes due to disability and length of hospital stay, not by disability or length of hospital stay itself.

There were several limitations to the present study. First, this study did not adjust for multiple morbidities and chronic diseases of occupationally injured workers; therefore, further studies need to consider workers with multiple disabilities. Second, medical information, such as cause of injury and medical expenditures, were not adjusted in this analysis because those variables were not available in PSWCI. Third, 24% of respondents did not complete all five years of follow-up after completing workers’ compensation care. There may be heterogeneity in general life satisfaction, socioeconomic status, and health status between respondents who have completed and those who did not. Lastly, this study was not based on a causal relationship model; therefore, we could not determine causal relationships between the variables.

## 5. Conclusions

To our knowledge, this is the first panel study to examine sex differences in factors associated with general life satisfaction among occupationally injured workers. The findings showed sex differences in factors related to general life satisfaction, especially in marital status and socioeconomic status, which highlights the need for sex-specific intervention programs for occupationally injured workers. Employers, healthcare providers, and other stakeholders need to pay attention to vulnerable groups, such as middle-aged men; men who are divorced, separated, or widowed; and men and women who do not return to work, and investigate the financial support level most appropriate for injured workers.

## Figures and Tables

**Table 1:** General characteristics of study subjects by survey year.

Variable	Subcategory	Time After Treatment													
		After 1 Year (First Wave)							After 6 Years (Fifth Wave)						
		Female		Male		Total		*p*-Value	Female		Male		Total		*p*-Value
		*n*	%	*n*	%	*n*	%		*n*	%	*n*	%	*n*	%	
Age (years)	29 or younger	11	4.2	75	6.0	86	5.7	<0.001	6	2.3	24	1.9	30	2.0	<0.001
	30 to 39	15	5.7	196	15.6	211	13.9		10	3.8	140	11.2	150	9.9	
	40 to 49	51	19.5	321	25.6	372	24.6		31	11.9	280	22.3	311	20.5	
	50 to 59	112	42.9	442	35.3	554	36.6		100	38.3	403	32.2	503	33.2	
	60 or older	72	27.6	219	17.5	291	19.2		114	43.7	406	32.4	520	34.3	
Marital status	Never married	16	6.1	195	15.6	211	13.9	<0.001	13	5.0	166	13.2	179	11.8	<0.001
	Divorced, separated, or widowed	79	30.3	125	10.0	204	13.5		86	33.0	150	12.0	236	15.6	
	Married	166	63.6	933	74.5	1099	72.6		162	62.1	937	74.8	1099	72.6	
Education level	Middle school or lower	149	57.1	470	37.5	619	40.9	<0.001	149	57.1	466	37.2	615	40.6	<0.001
	High school graduate	85	32.6	581	46.4	666	44.0		84	32.2	580	46.3	664	43.9	
	College or higher	27	10.3	202	16.1	229	15.1		28	10.7	207	16.5	235	15.5	
Return to work	Unemployed	101	38.7	333	26.6	434	28.7	<0.001	80	30.7	210	16.8	290	19.2	<0.001
	Different workplace	75	28.7	454	36.2	529	34.9		130	49.8	691	55.1	821	54.2	
	Pre-injury workplace	85	32.6	466	37.2	551	36.4		51	19.5	352	28.1	403	26.6	
Personal labor income	1st quartile (lowest)	63	24.1	321	25.6	384	25.4	<0.001	111	42.5	275	21.9	386	25.5	<0.001
	2nd quartile	122	46.7	276	22.0	398	26.3		126	48.3	250	20.0	376	24.8	
	3rd quartile	61	23.4	301	24.0	362	23.9		18	6.9	356	28.4	374	24.7	
	4th quartile (highest)	15	5.7	355	28.3	370	24.4		6	2.3	372	29.7	378	25.0	
NBLA recipient	No	255	97.7	1232	98.3	1487	98.2	0.446	258	98.9	1244	99.3	1502	99.2	0.445
	Yes	6	2.3	21	1.7	27	1.8		3	1.1	9	0.7	12	0.8	
Self-rated health	Very bad	18	6.9	79	6.3	97	6.4	0.855	14	5.4	74	5.9	88	5.8	0.015
	Bad	105	40.2	475	37.9	580	38.3		99	37.9	351	28.0	450	29.7	
	Good	127	48.7	646	51.6	773	51.1		137	52.5	757	60.4	894	59.0	
	Very good	11	4.2	53	4.2	64	4.2		11	4.2	71	5.7	82	5.4	
Length of hospital stay (days)	1 to 364	242	92.7	1114	88.9	1356	89.6	0.138	242	92.7	1114	88.9	1356	89.6	0.138
	365 to 729	16	6.1	104	8.3	120	7.9		16	6.1	104	8.3	120	7.9	
	730 or longer	3	1.1	35	2.8	38	2.5		3	1.1	35	2.8	38	2.5	
Self-esteem	Normal	232	88.9	1120	89.4	1352	89.3	0.813	232	88.9	1128	90.0	1360	89.8	0.581
	Low	29	11.1	133	10.6	162	10.7		29	11.1	125	10.0	154	10.2	
	**Range**	**Mean**	**SD**	**Mean**	**SD**	**Mean**	**SD**		**Mean**	**SD**	**Mean**	**SD**	**Mean**	**SD**	
Task performance ^†^	(min = 0; max = 10)	6.3	2.4	6.5	2.4	6.4	2.4	0.405	7.3	2.3	7.5	2.2	7.5	2.2	0.209
Disability grade ^†^	(min = 1; max = 15)	12.6	2.3	11.9	2.7	12.1	2.6	<0.001	12.6	2.3	11.9	2.7	12.1	2.6	<0.001
Self-efficacy ^†^	(min = 34; max = 111)	76.0	9.7	78.4	10.0	78.0	10.0	<0.001	75.7	9.7	78.4	9.6	77.9	9.7	<0.001
Life satisfaction ^†^	(min = 1; max = 5)	3.2	0.5	3.3	0.5	3.3	0.5	0.178	3.4	0.5	3.4	0.5	3.4	0.5	0.833

Number of study subjects (total = 1,514; male = 1,253; female = 261); ^†^ Higher values represent better conditions. NBLA, National Basic Livelihood Act; SD, standard deviation.

**Table 2:** Factors associated with general life satisfaction among occupationally injured workers in Korea.

Variable	Subcategory	Female				Male				Total			
		Coeff	SE	95% CI		Coeff	SE	95% CI		Coeff	SE	95% CI	
Age (years)	29 or younger	ref				ref				ref			
	30 to 39	0.067	0.039	−0.010	0.143	−0.012	0.012	−0.036	0.012	−0.001	0.012	−0.025	0.023
	40 to 49	0.089	0.047	−0.003	0.181	−0.033 *	0.013	−0.059	−0.008	−0.020	0.013	−0.046	0.006
	50 to 59	0.102 *	0.048	0.008	0.197	−0.017	0.013	−0.043	0.009	−0.004	0.014	−0.031	0.023
	60 or older	0.116 *	0.050	0.018	0.215	−0.008	0.014	−0.035	0.020	0.006	0.014	−0.022	0.035
Sex	Female	N/A				N/A				ref			
	Male									−0.028 ***	0.007	−0.042	−0.014
Marital status	Never married	Ref				ref				ref			
	Divorced, separated, or widowed	−0.038	0.038	−0.113	0.037	−0.038 **	0.012	−0.061	−0.014	−0.031 **	0.011	−0.052	−0.009
	Married	0.003	0.036	−0.066	0.073	0.032 ***	0.009	0.015	0.049	0.030 **	0.009	0.013	0.047
Education level	Middle school or lower	ref				ref				ref			
	High school graduate	0.011	0.015	−0.019	0.042	0.014 *	0.006	0.002	0.026	0.014 *	0.006	0.003	0.026
	College or higher	0.072 **	0.028	0.018	0.127	0.021 *	0.009	0.004	0.037	0.026 **	0.008	0.010	0.042
Return to work	Unemployed	ref				ref				ref			
	Different workplace	−0.001	0.010	−0.020	0.018	0.015 *	0.006	0.004	0.027	0.012 *	0.005	0.003	0.022
	Pre-injury workplace	0.029 *	0.013	0.005	0.054	0.034 ***	0.007	0.021	0.047	0.033 ***	0.006	0.022	0.045
Personal labor income	1st quartile (lowest)	ref				ref				ref			
	2nd quartile	0.006	0.009	−0.012	0.023	0.015 **	0.006	0.004	0.026	0.012 **	0.005	0.003	0.022
	3rd quartile	−0.012	0.014	−0.038	0.015	0.022 ***	0.006	0.011	0.033	0.016 **	0.005	0.007	0.026
	4th quartile (highest)	0.033	0.023	−0.012	0.079	0.047 ***	0.006	0.035	0.058	0.042 ***	0.006	0.031	0.053
NBLA recipients	No	ref				ref				ref			
	Yes	−0.064	0.035	−0.133	0.004	−0.069 *	0.034	−0.136	−0.002	−0.068 *	0.028	−0.122	−0.013
Self-rated health	Very bad	ref				ref				ref			
	Bad	0.009	0.022	−0.035	0.053	0.030 **	0.010	0.012	0.049	0.025 **	0.009	0.008	0.042
	Good	0.037	0.024	−0.011	0.084	0.052 ***	0.010	0.032	0.071	0.047 ***	0.009	0.029	0.065
	Very good	0.073 *	0.030	0.014	0.133	0.056 ***	0.012	0.031	0.080	0.055 ***	0.011	0.033	0.078
Task performance ^†^	(min = 0; max = 10)	0.008 **	0.003	0.003	0.013	0.002 *	0.001	0.000	0.005	0.004 ***	0.001	0.002	0.006
Disability grade ^†^	(min = 1; max = 15)	0.003	0.003	−0.003	0.010	0.000	0.001	−0.002	0.002	0.001	0.001	−0.001	0.003
Length of hospital stay (days)	1 to 364	ref				ref				ref			
	365 to 729	−0.031	0.024	−0.078	0.016	−0.003	0.010	−0.023	0.017	−0.006	0.010	−0.025	0.013
	730 or longer	0.034	0.083	−0.129	0.198	−0.014	0.019	−0.051	0.024	−0.008	0.019	−0.045	0.028
Self-esteem	Normal	ref				ref				ref			
	Low	−0.042 **	0.014	−0.069	-0.016	−0.046 ***	0.007	−0.060	−0.031	−0.045 ***	0.006	−0.057	−0.032
Self-efficacy ^†^	(min = 34; max = 111)	0.003 ***	0.001	0.002	0.004	0.003 ***	0.000	0.002	0.003	0.003 ***	0.000	0.002	0.003
Time after treatment (years)	1	ref				ref				ref			
	2	0.003	0.010	−0.016	0.022	−0.002	0.004	−0.010	0.006	−0.001	0.004	−0.008	0.007
	3	0.015	0.011	−0.006	0.035	0.009 *	0.004	0.000	0.017	0.010 *	0.004	0.002	0.018
	4	0.028 *	0.011	0.006	0.050	0.019 ***	0.005	0.010	0.028	0.021 ***	0.004	0.013	0.029
	5	0.033 **	0.011	0.012	0.054	0.024 ***	0.005	0.015	0.034	0.026 ***	0.004	0.018	0.035

^†^ Higher values represent better conditions. *** *p* < 0.001; ** *p* < 0.01; * *p* < 0.05. SE, standard error; coeff, coefficient; CI, confidence interval; NBLA, National Basic Livelihood Act; ref, reference; N/A, not applicable.
